# Supplementary material for: Circulating neutrophils from patients with early breast cancer have distinct subtype-dependent phenotypes
Source: Breast Cancer Res. 2023 Oct 19;25:125. doi: 10.1186/s13058-023-01707-3 (PMC10588170; doi:10.1186/s13058-023-01707-3)
Supplement: Supplementary file 1 — Additional file 1. Figure S1. Gating strategy to identifying human neutrophils via flow cytometry. [file 13058_2023_1707_MOESM1_ESM.docx]

**Supplementary Figure 1.**

**Gating strategy to identifying human neutrophils via flow cytometry.**


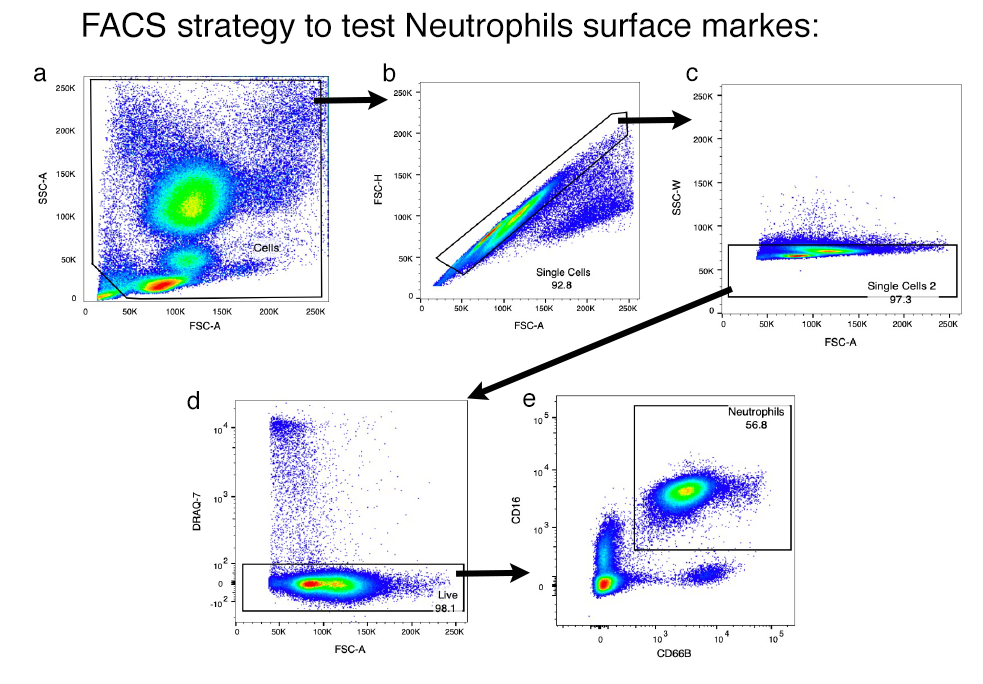


**Gating strategy to identifying human neutrophils via flow cytometry.** **a:** Gating on cells based on side scatter area (SCC-A on y-axis) and forward scatter (FSC-A on x- axis); **b:** Gating on single cells based on forward scatter-height (FSC-H) on y-axis to exclude cell doublets; **c:** Gating on single cells based on side scatter-width (SCC-W) on y-axis to exclude cell doublets; **d**: Gating on live cells which are negative for live/dead dye DRAQ7 on y-axis; **e:** Gating on neutrophils which are positive for surface markers CD16 and CD66b).
