# Supplementary material for: Circulating neutrophils from patients with early breast cancer have distinct subtype-dependent phenotypes
Source: Breast Cancer Res. 2023 Oct 19;25:125. doi: 10.1186/s13058-023-01707-3 (PMC10588170; doi:10.1186/s13058-023-01707-3)
Supplement: Supplementary file 2 — Additional file 2. Figure S2. Neutrophils analysis in a MMTV-pyMT model of breast cancer. [file 13058_2023_1707_MOESM2_ESM.docx]

**Supplementary Figure 2**

**Neutrophils analysis in a MMTV-pyMT model of breast cancer**

**
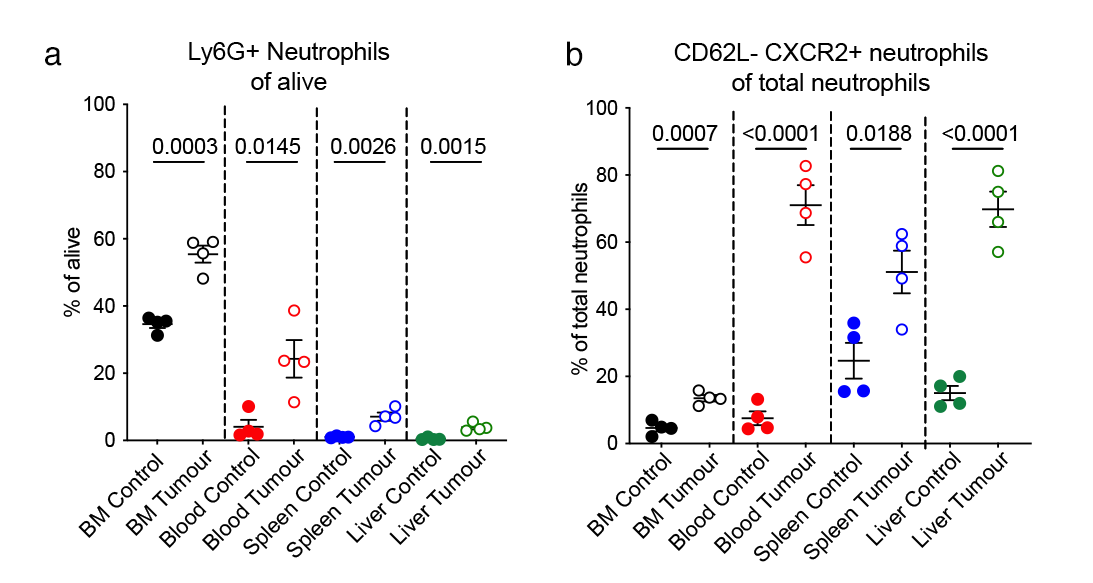
**

**Neutrophils analysis in a preclinical model of breast cancer. a**. Flow cytometry showing neutrophils (LY6G^+^ CD11b^+^) in the bone marrow (BM), blood, spleen, liver and lungs of tumour-bearing mice and healthy controls. Each dot represents one mouse. Data are shown as mean ± standard deviation (SD). Statistical analysis by two-sided t-test. **b**. Flow cytometry showing proportion of CD62L-CXCR2+ of total neutrophils (LY6G^+^CD11b^+^) in the BM, blood, spleen and liver of tumour-bearing mice and healthy controls. Each dot represents one mouse. Data are shown as mean ± standard deviation (SD). Statistical analysis by two-sided t-test.
