# Supplementary material for: Circulating neutrophils from patients with early breast cancer have distinct subtype-dependent phenotypes
Source: Breast Cancer Res. 2023 Oct 19;25:125. doi: 10.1186/s13058-023-01707-3 (PMC10588170; doi:10.1186/s13058-023-01707-3)
Supplement: Supplementary file 3 — Additional file 3. Table S1. Table of characteristics for patients with breast cancer. [file 13058_2023_1707_MOESM3_ESM.docx]

**Supplementary Table 1**

**Table of characteristics for patients with breast cancer**

| Patient details | Age | Menopausal status | Tumour size (mm) | Tumour Grade | Histology | Nodal Status | ER status | PR status | HER2 status | Pre chemo/Surgery | Medical conditions | Medication |
| --- | --- | --- | --- | --- | --- | --- | --- | --- | --- | --- | --- | --- |
| P01 | 59 | Post | 20 + 26 | 2 | IDC | 7/15 | 8 | 8 | Neg | Pre-surgery | Asthma | Salbutamol |
| P02 | 60 | Post | 19 | 3 | IDC | 0/3 | 8 | 6 | Neg | Pre-surgery | Asthma  Hypothyroidism | Amlodipine  Atenolol  Ramipril |
| P03 | 51 | Post | 20 | 2 | IDC | 15/30 | 8 | 5 | Neg | Pre-surgery | Nil | Nil |
| P04 | 52 | Post | 16 and  31 | 1 and  2 | IDC and  IMC | 0/3 + 0/1 | 8  8 | 7  7 | Neg  Neg | Pre-surgery | Nil | Nil |
| P05 | 73 | Post | 23 | 2 | IDC | 0/5 | 8 | 8 | Neg | Pre-surgery | Glaucoma | Latanoprost  Brinzolamide |
| P06 | 63 | Post | 21 | 2 | IDC | 0/3 | 8 | 8 | Neg | Pre-surgery | Asthma | Salbutamol  Beclomethasone |
| P07 | 58 | Post | 16 | 3 | IDC | 0/2 | 8 | 8 | Neg | Pre-surgery | High cholesterol | Atorvastatin |
| P08 | 54 | Post | 24 | 2 | ILC | 0/1 | 8 | 5 | Neg | Pre-surgery | Depression  Reflux | Citalopram  Omeprazole |
| P09 | 50 | Post | 20 | 3 | IDC | 16/17 | 8 | 8 | Neg | Pre-surgery | Nil | Nil |
| P10 | 69 | Post | 18 | 2 | ILC | 0/2 | 7 | 8 | Neg | Pre-surgery | Nil | Nil |
| P11 | 51 | Post | 9 | 2 | ILC | 0/2 | 8 | 8 | Neg | Pre-surgery | Nil | Nil |
| P12 | 42 | Pre | 15 | 2 | IDC | 0/6 | 8 | 8 | Neg | Pre-surgery | Nil | Nil |
| P13 | 44 | Pre | 20 | 3 | IDC | 0/7 | 0 | 0 | Neg | Pre-chemo | Rosacea | Mebeverine |
| P14 | 51 | Pre | 30 + 19 | 2 | IDC | 11/23 | 8 | 8 | Neg | Pre-surgery | Nil | Nil |
| P15 | 46 | Pre | 20 | 3 | IDC | 3/14 | 8 | 0 | Neg | Pre-chemo | Nil | Marina Coil |
| P16 | 31 | Pre | 25 | 3 | IDC | 0/4 | 8 | 3 | Neg | Pre-surgery | Nil | Nil |
| P17 | 55 | Post | 21 | 1 | IDC | 1/1 | 8 | 7 | Neg | Pre-surgery | Hypertension | Amlodipine |
| P18 | 46 | Pre | 34 | 3 | IDC | 0/1 | 0 | 0 | Neg | Pre-chemo | Asthma | Salbutamol PRN |
| P19 | 57 | Post | 33 | 3 | IDC | 0/1 | 0 | 0 | Neg | Pre-chemo | Hay fever | Piriton PRN |
| P20 | 55 | Post | 27 | 3 | IDC | 1/1 | 0 | 0 | Pos | Pre-chemo | Hay fever | Nil |
| P21 | 56 | Post | 20 | 2 | IDC | 0/5 | 8 | 4 | Neg | Pre-surgery | Nil | Nil |
| P22 | 52 | Pre | 23 | 3 | IDC | 0 | 0 | 4 | Neg | Pre-chemo | Nil | Nil |
| P23 | 55 | Post | 120 x 100 | 2 | IDC | 1 | 0 | 0 | Pos | Pre-chemo | Nil | Nil |
| P24 | 66 | Post | 25 | 2 | IDC | 0 | 8 | 7 | Pos | Pre-chemo | Nil | Nil |
| P25 | 63 | Post | 22 | 3 | IDC | 0 | 0 | 2 | Pos | Pre-chemo | Nil | Nil |
| P26 | 33 | Pre | 76 | 3 | IDC | 0 | 0 | 0 | Pos | Pre-chemo | Nil | Nil |
| P27 | 50 | Pre | 28 | 2 | IDC | 0 | 0 | 0 | Neg | Pre-chemo | Nil | Nil |
| P28 | 74 | Post | 36 | 3 | IDC | 7 | 8 | 7 | Pos | Pre-chemo | Nil | Nil |
| P29 | 38 | Pre | 108 | 2 | IDC | 1 | 8 | 8 | Neg | Pre-surgery | Nil | Nil |
| P30 | 39 | Pre | 64 | 2 | IDC | 0 | 7 | 6 | Pos | Pre-chemo | Nil | Nil |
| P31 | 30 | Pre | 29 | 3 | IDC | 0 | 0 | 0 | Neg | Pre-chemo | Nil | Nil |
| P32 | 55 | Post | 37 | 2 | IDC | 0 | 8 | 7 | Neg | Pre-surgery | Nil | Nil |
| P33 | 44 | Pre | 21 | 3 | IDC | 0 | 2 | 0 | Neg | Pre-chemo | Nil | Nil |
| P34 | 35 | Pre | 33 | 2 | IDC | 0 | 7 | 7 | Pos | Pre-chemo | Nil | Nil |
| P35 | 43 | Pre | 36 | 3 | IDC | 0 | 8 | 8 | Pos | Pre-chemo | Nil | Nil |
| P36 | 55 | Post | 52 | 2 | IDC | +3 | 8 | 0 | Pos | Pre-chemo | Nil | Nil |
| P37 | 51 | Pre | 75 | 22 | IDC | +3 | 3 | 0 | Pos | Pre-chemo | Difficulty sleeping | Zopiclone |
| P38 | 42 | Pre | 47 | 3 | IDC | +3 | 0 | 0 | Pos | Pre-chemo | Nil | Nil |
| P39 | 57 | Pos | 27 | 2 | IDC | 0 | 8 | 6 | Pos | Pre-chemo | Nil | Nil |
| P40 | 51 | Post | 20 | 2 | IDC | 0 | 8 | 8 | Neg | Pre-surgery | Nil | Nil |
| P41 | 35 | Pre | 22 | 3 | IDC | 0 | 2 | 0 | Neg | Pre-chemo | Nil | Nil |
| P42 | 68 | Post | 30 | 3 | IDC | 0 | 0 | 2 | Pos | Pre-chemo | Nil | Nil |
| P43 | 51 | Pre | 21 | 1 | IDC | 0 | 8 | 8 | Neg | Pre-surgery | Nil | Nil |
| P44 | 69 | Post | 25 | 2 | IDC | 0 | 0 | 0 | Pos | Pre-chemo | Nil | Nil |

Table 1a: Patient characteristics for Patient P01 – P44 including patient age, menopausal status, tumour size, histology, grade, nodal status, medical conditions, medication and time of blood taken (surgery/ pre chemotherapy). Note abbreviations for Pre-menopausal (Pre) and Post-menopausal (Post) and blood taken Pre-surgery or Pre-chemotherapy (Pre-chemo). Histology of breast tumour classified as Invasive ductal carcinoma (IDC) or Invasive lobular carcinoma (ILC). HER2 status of tumour classified as Positive (Pos) or Negative (Neg).

**Table of characteristics for Healthy Volunteers (HVs) paired with breast cancer patients**

| Patient details | Age | Menopausal status | Medical conditions | Medication |
| --- | --- | --- | --- | --- |
| HV01 | 56 | Post | Nil | Nil |
| HV02 | 53 | Post | Nil | Nil |
| HV03 | 48 | Post | Nil | Nil |
| HV04 | 53 | Post | Nil | Nil |
| HV06 | 61 | Post | Nil | Nil |
| HV07 | 52 | Post | Nil | Nil |
| HV08 | 53 | Post | Nil | Nil |
| HV09 | 48 | Post | Hypertension | Amlodipine  Bendoflumethiazide |
| HV10 | 58 | Post | Nil | Nil |
| HV11 | 53 | Post | Nil | Nil |
| HV12 | 41 | Pre | Nil | Nil |
| HV13 | 51 | Pre | Nil | Nil |
| HV14a | 47 | Pre | Nil | Nil |
| HV14b | 53 | Post | Nil | Nil |
| HV15 | 41 | Pre | Nil | Nil |
| HV16 | 33 | Pre | Nil | Marina Coil |
| HV17 | 53 | Post | Nil | Nil |
| HV18 | 46 | Pre | Nil | Nil |
| HV19 | 59 | Post | Nil | Nil |
| HV20 | 56 | Post | Hay fever | Nexium |
| HV21 | 55 | Post | Nil | Nil |
| HV22 | 46 | Pre | Nil | Nil |
| HV23 | 57 | Post | Nil | Nil |
| HV24 | 59 | Post | Nil | Nil |
| HV25 | 52 | Post | Nil | Nil |
| HV26 | 28 | Pre | Nil | Nil |
| HV27 | 48 | Pre | Nil | Nil |
| HV28 | 61 | Post | Hypertension  Reflux | Omeprazole  Ramipril  Atenolol  Indapamide |
| HV29 | 36 | Pre | Anxiety  Depression | Sertraline |
| HV30 | 42 | Pre | Nil | Nil |
| HV31 | 30 | Pre | Nil | Nil |
| HV32 | 57 | Post | Nil | Nil |
| HV33 | 37 | Pre | Nil | Nil |
| HV34 | 34 | Pre | Nil | Nil |
| HV35 | 35 | Pre | Nil | Nil |
| HV36 | 52 | Post | Nil | Nil |
| HV37 | 43 | Pre | Nil | Nil |
| HV38 | 54 | Pre | Nil | Nil |
| HV39 | 56 | Post | Nil | Nil |
| HV40 | 60 | Post | Mild asthma | Salbutamol PRN |
| HV41 | 29 | Pre | Nil | Nil |
| HV42 | 62 | Post | Nil | Nil |
| HV43 | 36 | Pre | Nil | Nil |
| HV44 | 55 | Post | Nil | Nil |

Table 1b: Characteristics of healthy volunteers (HV01-HV44) who were used for pairing with patients with breast cancer. HV characteristics include age, menopausal status, medical conditions and medication. Note for the purposes of flow cytometry analysis HV14a was used as pairing with P14.
