# Supplementary material for: Circulating neutrophils from patients with early breast cancer have distinct subtype-dependent phenotypes
Source: Breast Cancer Res. 2023 Oct 19;25:125. doi: 10.1186/s13058-023-01707-3 (PMC10588170; doi:10.1186/s13058-023-01707-3)
Supplement: Supplementary file 4 — Additional file 4. Table S2. a. Table of characteristics for patients with benign breast disease. b. Table of characteristics for HVs paired with patients with benign breast disease. [file 13058_2023_1707_MOESM4_ESM.docx]

**Supplementary Table 2**

**a. Table of characteristics for patients with benign breast disease**

| Patient details | Age | Menopausal status | Benign breast disease histology | Lesion size (mm) | Medical conditions | Medication |
| --- | --- | --- | --- | --- | --- | --- |
| B01 | 31 | Pre | Fibroadenoma | 36 | None | Combined oral contraceptive |
| B02 | 25 | Pre | Fibroadenoma | 16 | Nil | Nil |
| B03 | 24 | Pre | Fibroadenoma | 21 | Nil | Nil |
| B04 | 24 | Pre | Fibroadenoma | 46 | Nil | Nil |
| B05 | 23 | Pre | Fibroadenoma | 46 | Nil | Nil |
| B06 | 33 | Pre | Fibroadenoma | 50 | Nil | Nil |
| B07 | 31 | Pre | Fibroadenoma | 50 | Nil | Nil |
| B08 | 18 | Pre | Fibroadenoma | 48 | Nil | Nil |
| B09 | 18 | Pre | Fibroadenoma | 40 | Nil | Nil |

##### Characteristics of patients with benign breast disease for patients B01-B09 including age, menopausal status, histology of benign breast disease, size of breast mass, medical conditions and medication.

**b. Table of characteristics for HVs paired with patients with benign breast disease**

| Patient details | Age | Menopausal status | Medical conditions | Medication |
| --- | --- | --- | --- | --- |
| HVB01 | 24 | Pre | Anxiety | Propanolol  Sertraline  Marina coil |
| HVB02 | 30 | Pre | Nil | Nil |
| HVB03 | 28 | Pre | Nil | Nil |
| HVB04 | 30 | Pre | Nil | Nil |
| HVB05 | 24 | Pre | Nil | Nil |
| HVB06 | 30 | Pre | Nil | Cerazette (Progesterone) |
| HVB07 | 18 | Pre | Nil | Nil |
| HVB08 |  | Pre | Nil | Nil |
| HVB09 | 18 | Pre | Nil | Nil |

##### Characteristics of healthy volunteers HVB01-HVB09 which were used for pairing with the patients with benign breast disease. HV characteristics include age, menopausal status, medical conditions and medication
