# Supplementary material for: Circulating neutrophils from patients with early breast cancer have distinct subtype-dependent phenotypes
Source: Breast Cancer Res. 2023 Oct 19;25:125. doi: 10.1186/s13058-023-01707-3 (PMC10588170; doi:10.1186/s13058-023-01707-3)
Supplement: Supplementary file 5 — Additional file 5. Table S3. Outline of inclusion and exclusion criteria for human pilot study. [file 13058_2023_1707_MOESM5_ESM.docx]

**Supplementary Table 3**

**Outline of inclusion and exclusion criteria for human pilot study**

|  | **Pre-surgery (including pre neoadjuvant chemotherapy)**  **44 patients** | **Benign breast disease**  **9 patients** | |
| --- | --- | --- | --- |
| Receptor status | HR+ (ER+/PR+)  HER2+ve  Triple negative | N/A | |
| Treatment regimen | None | N/A |  |
| Exclusion Criteria | - Drugs: NSAIDs, Steroids, Antibiotics, Carbimazole, Phenytoin, immunomodulating drugs - Acute infection - Chronic infection (TB, Hep B/C)/chronic inflammatory conditions/ autoimmune conditions/ immunodeficiency disorders including HIV | | |
| Timing of blood samples | - On the day of surgery - Prior to neoadjuvant chemotherapy | - On the day of surgery | |

##### Table 3: Outline of inclusion and exclusion criteria for selection of patients with breast cancer, patients with benign breast disease and their respective paired HVs
