# Supplementary material for: Circulating neutrophils from patients with early breast cancer have distinct subtype-dependent phenotypes
Source: Breast Cancer Res. 2023 Oct 19;25:125. doi: 10.1186/s13058-023-01707-3 (PMC10588170; doi:10.1186/s13058-023-01707-3)
Supplement: Supplementary file 6 — Additional file 6. Figure S3. Measurement of circulating neutrophils life span ex vivo. [file 13058_2023_1707_MOESM6_ESM.docx]

**Supplementary Figure 3**

**Measurement of circulating neutrophils life span *ex vivo*.**


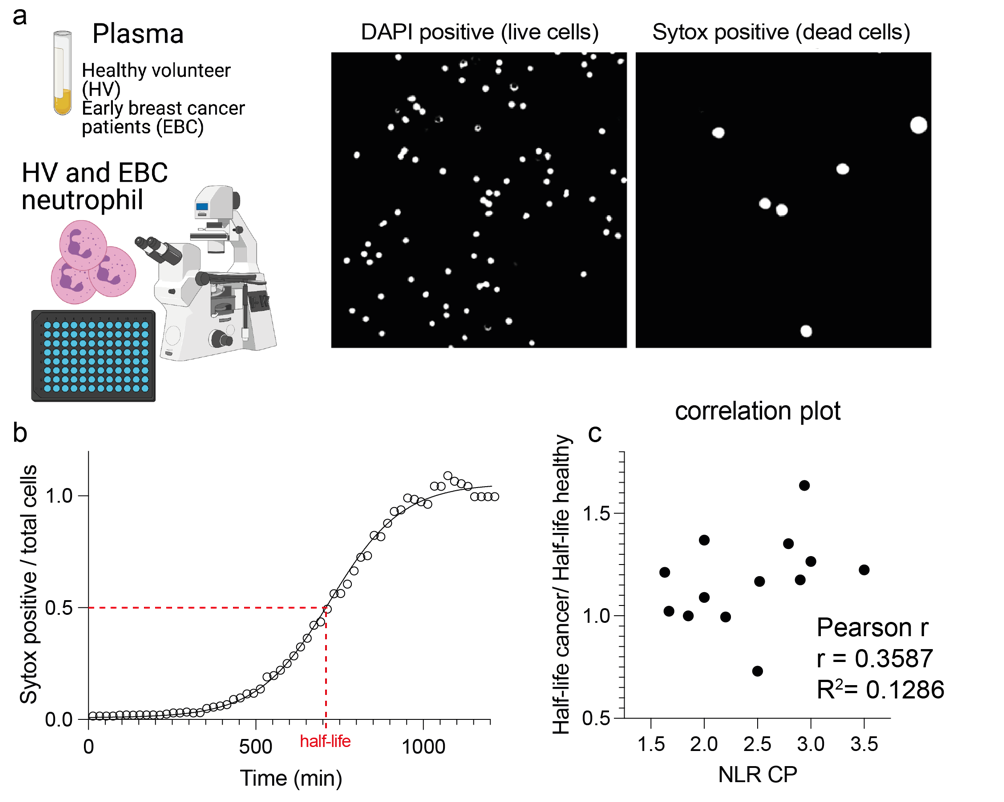


**Measurement of circulating neutrophils life span *ex vivo.* a**. Scheme of the experimental set-up for the live cell imaging and half-life quantification. Example of live and dead cells identified by intensity-based thresholding at a specific timepoint. **b**. representative live curve of number of dead cells over total cells of a specific field of view over time and the half-live (dotted red line) representing the time at which 50% of the total cells are dead. **c**. Correlation analysis of the neutrophil to lymphocyte ratio measured in EBC patients and the fold change in the corresponding half-life value compared to the one obtained for the matching HV.
