# Supplementary material for: Circulating neutrophils from patients with early breast cancer have distinct subtype-dependent phenotypes
Source: Breast Cancer Res. 2023 Oct 19;25:125. doi: 10.1186/s13058-023-01707-3 (PMC10588170; doi:10.1186/s13058-023-01707-3)
Supplement: Supplementary file 7 — Additional file 7. Figure S4. Measurements of phosphorylated target peptides specific to either serine/threonine or tyrosine kinases. [file 13058_2023_1707_MOESM7_ESM.docx]

**Supplementary Figure 4**

**Measurements of phosphorylated target peptides specific to either serine/threonine or tyrosine kinases.**

**a. Example of luminescent readout**


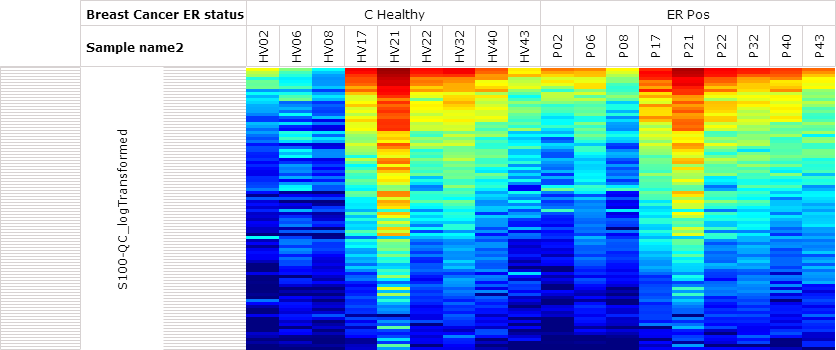


Log-fold change in

**b. Serine Threonine kinase (STK)** **c.** **Phosphotyrosine kinase (PTK)**
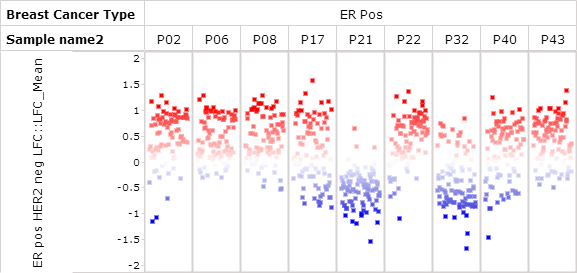


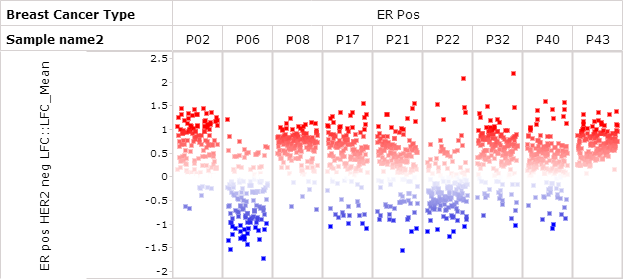


a. Heatmap to show an overview of peptides which have been phosphorylated by kinases within the neutrophil lysates in HVs (represented by column C Healthy) and patients with HR-positive HER2 negative breast cancer (represented by ER pos column). The sample name 2 row refers to the individual patients (letter P in front of numbers) and paired HV in front of numbers. Data represented prior to normalisation to accommodate for variability due to experiments done on different days

b-c Log-fold change (LFC) in phosphorylated peptides in patients with breast cancer compared to paired HVs (individual patients are represented by different columns) for STK (b) and PTK (c) family of kinases
