# Supplementary material for: Circulating neutrophils from patients with early breast cancer have distinct subtype-dependent phenotypes
Source: Breast Cancer Res. 2023 Oct 19;25:125. doi: 10.1186/s13058-023-01707-3 (PMC10588170; doi:10.1186/s13058-023-01707-3)
Supplement: Supplementary file 8 — Additional file 8. Figure S5. Principle Component Analysis in different subtypes compared to match HV. [file 13058_2023_1707_MOESM8_ESM.pdf]

Supplementary Figure 5

Principle Component Analysis in different subtypes compared to match HV.

a-d. Serine Threonine kinase (STK)

a

HR-positive HER2 negative pts Vs HVs

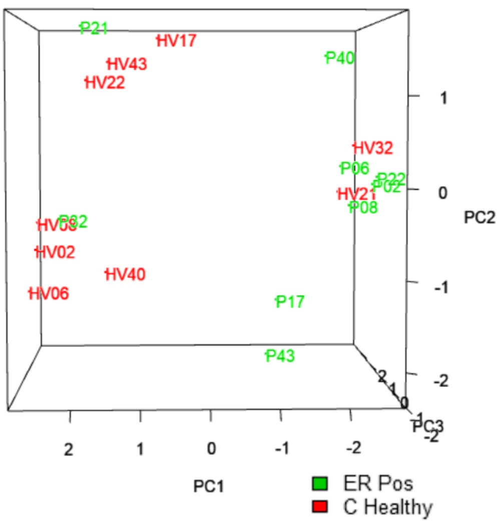

b

HR-positive HER2 positive pts Vs HVs

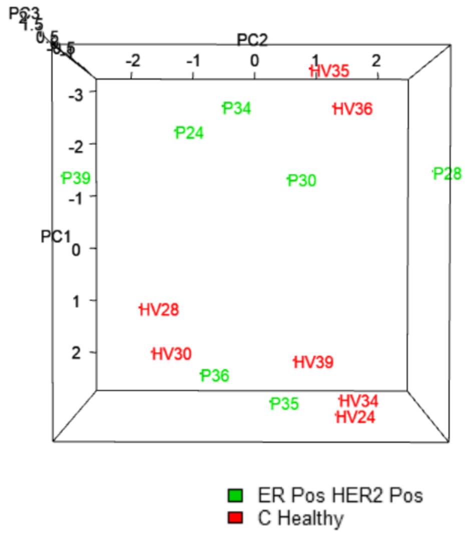

c

Triple negative pts Vs HVs

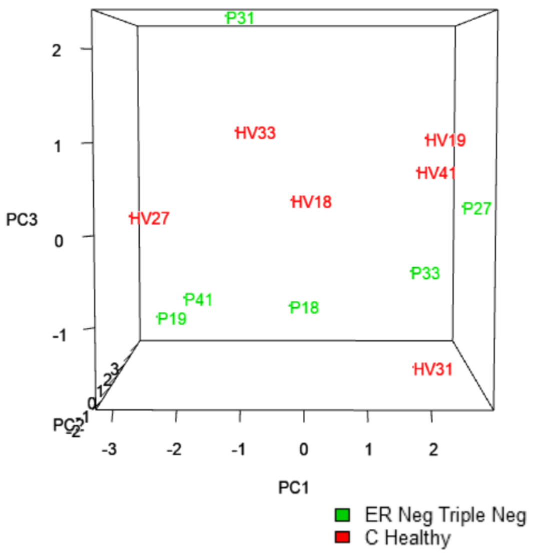

d

HR-negative HER2 positive pts Vs HVs

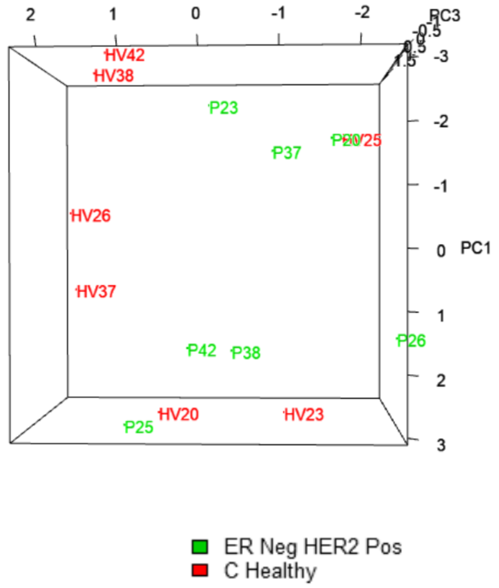

e-h. Phosphotyrosine kinase (PTK)

e

HR-positive HER2 negative patients Vs HVs

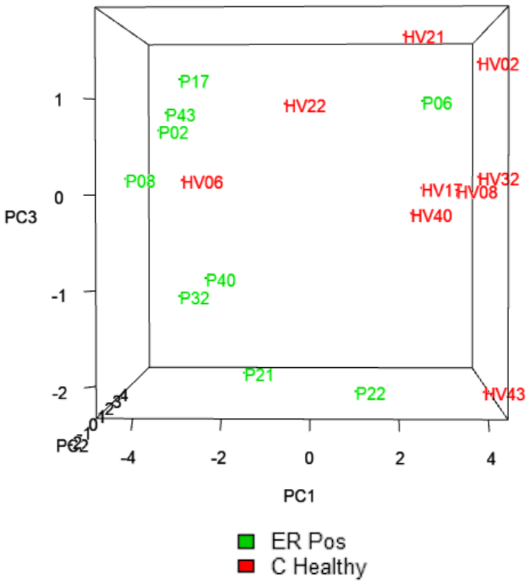

f

HR-positive HER2 positive patients Vs HVs

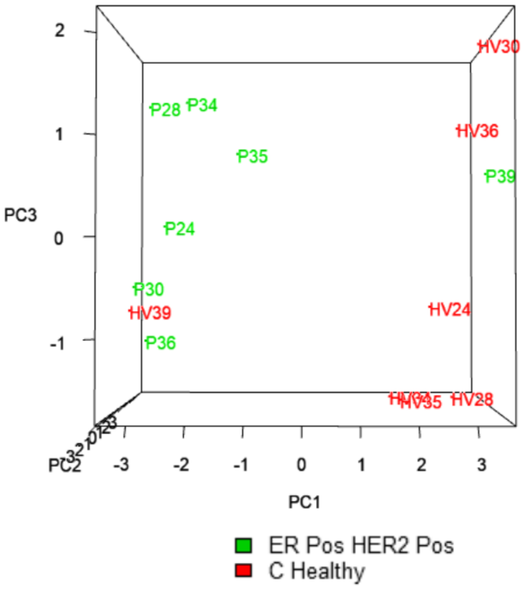

g

Triple negative patients Vs HVs

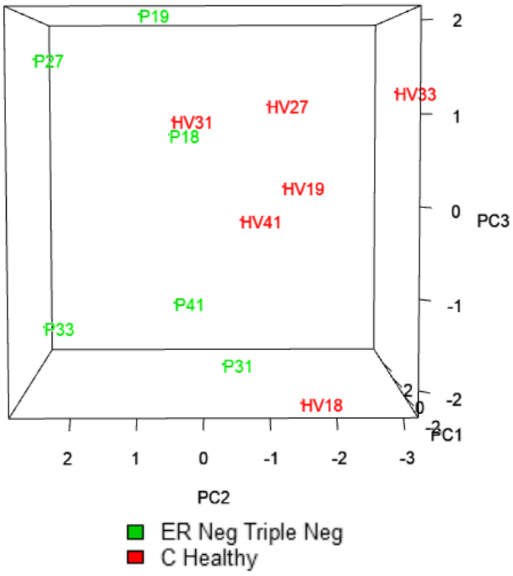

h

HR-negative HER2 positive patients Vs HVs

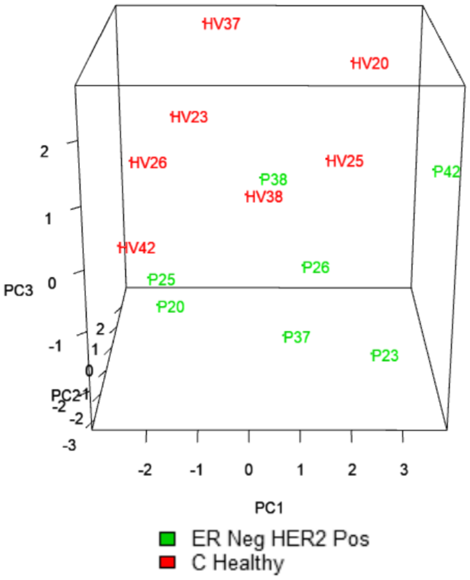

Principle Component Analysis in different subtypes compared to match HV.

a-d: Principal Component Analysis (PCA) of the different subtypes of breast cancer compared to HVs for the STK family of kinases. a. HR-positive HER2 negative breast cancer (represented by ER pos) and HVs (represented by C Healthy); b: patients with HR-positive HER2 positive breast cancer (represented by ER pos HER2 pos) and HVs (represented by C Healthy); c: patients with HR-negative HER2 positive breast cancer (represented by ER neg HER2 pos) and HVs (represented by C Healthy); d: patients with Triple negative breast cancer (represented by Triple neg) and HVs (represented by C Healthy).

e-h Principal Component Analysis (PCA) of the different subtypes of breast cancer compared to HVs for the PTK family of kinases. e: patients with HR-positive HER2 negative breast cancer (represented by ER pos) and HVs (represented by C Healthy); f: patients with HR-positive HER2 positive breast cancer (represented by ER pos HER2 pos) and HVs (represented by C Healthy); g: patients with HR-negative HER2 positive breast cancer (represented by ER neg HER2 pos) and HVs (represented by C Healthy); h: patients with Triple negative breast cancer (represented by Triple neg) and HVs (represented by C Healthy).
