# Supplementary material for: Circulating neutrophils from patients with early breast cancer have distinct subtype-dependent phenotypes
Source: Breast Cancer Res. 2023 Oct 19;25:125. doi: 10.1186/s13058-023-01707-3 (PMC10588170; doi:10.1186/s13058-023-01707-3)
Supplement: Supplementary file 10 — Additional file 10. R code of the machine learning algorithms for training the model according to the number of patients. [file 13058_2023_1707_MOESM10_ESM.pdf]

R code of the machine learning algorithms for training the model according to the number of patients.

```
## ------------------------------------------------------------------------------
## load required packages
## ------------------------------------------------------------------------------
require(clipr)
require(tidyr)
require(randomForest)
require(lightgbm)
require(caret)
require(shapviz)

## ------------------------------------------------------------------------------
## global variables
## ------------------------------------------------------------------------------
working_dir = "~/Desktop/tmp_ila"
setwd(working_dir)

# *** @user: choose the machine learning algorithm: 'rf' randomForest vs
# 'lgb' for lightgbm, for inbalanced data 'rf' is recommended
ml_model = 'rf'  # rf, lgb
# *** @user: choose the groups to do the classification for
groups_to_compare = 'benign_tumour'  #c('benign_tumour', 'erneg_pos', 'her2pos_neg', 'erpos_her2pos_vs_her2neg')

# random forest parameters
rf_nbr_var_sampled_for_each_split = 282 # since sparse data, we use all
# variables when constructing a tree
rf_nbr_trees = 1000 # default 500
rf_sampsize_for_both_classes = 5  # since we have 6 benign, 29 malign, we enforce
# balanced samples (bags) when creating a tree

# lightgbm (lgb) parameters
n_rounds = 100  # lgb variable

code_nas_as_zero <- TRUE
aggregate_ks <- FALSE  # multiply the two measures
create_varimportance_plots <- FALSE  # set to TRUE in order to get shapley and variable importance plots

## ------------------------------------------------------------------------------
## import data
## ------------------------------------------------------------------------------

# first time: import data from xls sheets (uncomment the below four lines )
# then save as RDS file
# from then onwards, just read the RDS files
# uncomment next four lines to import form speadsheet
# mfs <- read_clip_tbl()  # copy and paste tab “data_long_table_MFS” from excel file “Additional file 9”
# ks <- read_clip_tbl()  # copy and paste tab “data_long_table_KS” from excel file “Additional file 9”
# save(mfs, file="mfs.RData")
# save(ks, file="ks.RData")
# load('mfs.RData')
# load('ks.RData')

# quick look at data, number of observations, variables, etc
dim(mfs) # number of rows and columns
dim(ks)
head(mfs)
```

```
tail(mfs)
head(ks)
tail(ks)
colnames(ks)  # column names
colnames(mfs)
unique(ks$patient)
length(unique(ks$patient))  # 35 patients
unique(ks$Kinase.Name)  # unique Kinase.Name
length(unique(ks$Kinase.Name))  # 141 unique Kinase.Name for ks
length(unique(mfs$Kinase.Name))  # 141 unique Kinase.Name for ks
#table(ks[!duplicated(ks[, c('patient', 'subtype')]), # keep one line per patient
#          c('patient', 'subtype')]$subtype)  # frequencies of each subtype

## ------------------------------------------------------------------------------
## combine mfs and ks and pivot from long to wide form
## ------------------------------------------------------------------------------
# replace special characters in Kinase.Name (e.g. [ or /) with _br_ for [] and _bs_ for /
ks$Kinase.Name <- gsub("\\[", "_br_", gsub("\\]", "_br_", gsub("\\/", "_bs_", ks$Kinase.Name)))
mfs$Kinase.Name <- gsub("\\[", "_br_", gsub("\\]", "_br_", gsub("\\/", "_bs_", mfs$Kinase.Name)))

# for ks dataframe rename Kinase.Name to *_ks
ks$Kinase.Name = paste(ks$Kinase.Name, 'ks', sep="_")

# append ks long dataframe to mfs long dataframe, assign to dataframe mfs_ks
# first we need to rename Median.Final.score in mfs and Median.Kinase.Statistic
# into same column name
names(mfs)[names(mfs) == 'Median.Final.score'] <- 'value'
names(ks)[names(ks) == 'Median.Kinase.Statistic'] <- 'value'
mfs_ks = rbind(mfs, ks)  # append ks to mfs long df
dim(mfs_ks)  # has 882 rows

# save subtype column in separate df and remove from long df
subtype <- mfs_ks[c('patient', 'subtype')]
subtype <- subtype[!duplicated(subtype$patient), ]  # keep only one row per patient
mfs_ks <- mfs_ks[ , -which(names(mfs_ks) %in% c('subtype'))]

# pivot from long to wide form
df_wide <- reshape(mfs_ks, idvar = "patient", v.names="value",timevar = "Kinase.Name", direction = "wide")
dim(df_wide)  # 141 + 141 + 'patient' = 283 columns
colnames(df_wide) <- gsub("value.", "", colnames(df_wide))  # remove the 'value.' in front of colnames
df_wide <- df_wide[order(df_wide$patient),]  # ord3r acc to patient

# look at one patient sample (one row), only show variables for which data is available
names(df_wide[1, ])[!is.na(df_wide[1,])]  # one patient, data for which var available

# merge subtype back as additional 'target' or 'response', or 'y' variable
# (the one we aim to predict)
df_wide <- merge(df_wide, subtype, by='patient')
# View(df_wide)
#write.csv(df_wide, file='df_wide.csv', na='')

# 'JAK1~b'  posed a problem, remove tilde
colnames(df_wide) <- gsub("~", "__", colnames(df_wide))  # remove the 'value.' in front of colnames
colnames(df_wide)[order(colnames(df_wide))]

## ------------------------------------------------------------------------------
## explore variables (coverage, ranges, etc)
## ------------------------------------------------------------------------------
```

```
df_wide_predictors_only <-df_wide[, -which(colnames(df_wide) %in% c('subtype', 'patient'))]
names(df_wide_predictors_only)

n_obs_per_predictor = apply(df_wide_predictors_only, 2, FUN=function(y){sum(!is.na(y))})
n_obs_per_predictor <- n_obs_per_predictor[order(n_obs_per_predictor)]

table(n_obs_per_predictor)

## -----------------------------------------------------------------------------
## run DIY LOOCV
## -----------------------------------------------------------------------------
# due to lack of docu for lgb.cv we implement LOOCV ourselves

# define parameters
params = list(
  objective = 'binary',  # 'multiclass',
  metric = 'auc', #binary_error', #binary_logloss' #"multi_error",
  #num_class= 2,
  #min_data=1,
  #learning_rate = 1
  min_data_in_leaf = 2,  # this is an important parameter, default 20, but many of our var have only 1 or 2 observations
  # see https://lightgbm.readthedocs.io/en/latest/Parameters-Tuning.html
  return_cvbooster = TRUE  # for lgb.cv: return the booster for each loop
  # is_unbalance = TRUE  # https://github.com/microsoft/LightGBM/issues/695
  #scale_pos_weight = 29/31 #(6/29)
  # scale_pos_weight = (table(df_wide$subtype_simple)['zero']
  #                     / table(df_wide$subtype_simple)['one'])
)

# important https://stackoverflow.com/questions/46456381/cross-validation-in-lightgbm?utm_medium=organic&utm_source=google_rich_qa&utm_campaign=google_rich_qa
# In general, the purpose of CV is NOT to do hyperparameter optimisation. The purpose is to evaluate performance of model-building procedure
# here we don't do hyperparameter optim: we have params fixed

# -- prepare data
# pivot from long to wide form
df_wide <- reshape(mfs_ks, idvar = "patient", v.names="value",timevar = "Kinase.Name", direction = "wide")
dim(df_wide)  # 141 + 141 + 'patient' = 283 columns
colnames(df_wide) <- gsub("value.", "", colnames(df_wide))  # remove the 'value.' in front of colnames
df_wide <- df_wide[order(df_wide$patient),]  # order acc to patient

# look at one patient sample (one row), only show variables for which data is available
names(df_wide[1, ])[!is.na(df_wide[1,])]  # one patient, data for which var available

# merge subtype back as additional 'target' or 'response', or 'y' variable
# (the one we aim to predict)
df_wide <- merge(df_wide, subtype, by='patient')

# 'JAK1~b'  posed a problem, remove tilde
colnames(df_wide) <- gsub("~", "__", colnames(df_wide))  # remove the 'value.' in front of colnames
colnames(df_wide)[order(colnames(df_wide))]

switch(groups_to_compare,
  benign_tumour = {
    print('benign_tumour')
    # (1) merge all non-benign patients into group 'Bad'
    df_wide$subtype_simple <- ifelse(df_wide$subtype == 'Benign',"Good","Bad")
    # convert label to numeric: 1: Bad, 0:Good
    df_wide$subtype_simple <- -1 * (as.numeric(as.factor(df_wide$subtype_simple)) - 2)
```

```
  },
  erneg_pos = {
    print('erneg_pos')
    # (2) ER neg, ER pos
    # remove Benign
    df_wide = df_wide[!df_wide$subtype=='Benign',]
    df_wide$subtype_simple <- ifelse(grepl('ER neg HER2 pos', df_wide$subtype) |
                                     grepl('Triple neg', df_wide$subtype),"Neg","Pos")
    table(df_wide$subtype_simple)
    # convert label to numeric: 1: Pos, 0:Neg
    df_wide$subtype_simple <- 1 + (as.numeric(as.factor(df_wide$subtype_simple)) - 2)
  },
  her2pos_neg = {
    print('her2pos_neg')
    # # (3) HER2 pos in one group, HER2 neg and Triple neg in another
    table(df_wide$subtype_simple)  # frequencies of each subtype
    df_wide = df_wide[!df_wide$subtype=='Benign',]
    df_wide$subtype_simple <- ifelse(grepl('ER neg HER2 pos', df_wide$subtype) |
                                     grepl('ER pos HER2 pos', df_wide$subtype),"Pos","Neg")
    table(df_wide$subtype_simple)
    # convert label to numeric: 1: Pos, 0:Neg
    df_wide$subtype_simple <- 1 + (as.numeric(as.factor(df_wide$subtype_simple)) - 2)
  },
  erpos_her2pos_vs_her2neg = {
    print('erpos_her2pos_vs_her2neg')
    # (4) 'ER pos HER2 neg' vs 'ER pos HER2 pos'
    df_wide = df_wide[(!df_wide$subtype=='Benign') &
                        (!df_wide$subtype=='ER neg HER2 pos')
                      & (!df_wide$subtype=='Triple neg'),]
    df_wide$subtype_simple <- ifelse(grepl('ER pos HER2 pos', df_wide$subtype),"Pos","Neg")

    df_wide$subtype_simple <- 1 + (as.numeric(as.factor(df_wide$subtype_simple)) - 2)
  },
  print('check groups_to_compare')
)

if (ml_model == 'rf') {
  df_wide$subtype_simple <- as.factor(ifelse(df_wide$subtype_simple == 0, 'zero', 'one'))
}

# remove the unused, non-numeric, subtype column (otherwise matrix will treat all numbers as strings)
df_wide = df_wide[, -which(colnames(df_wide) %in% c('subtype', 'patient'))]

# replace all NAs with zero in df_wide
if (code_nas_as_zero) {
  df_wide[is.na(df_wide)] <- 0
}

# multiply _ks with their analogon var
if (aggregate_ks) {
  var_names <- colnames(df_wide_predictors_only)
  var_names <- var_names[!grepl('_ks', var_names)]
  for (vn in var_names) {
    df_wide[paste(vn, '_aggr', sep='')] = df_wide[vn] * df_wide[paste(vn,'_ks', sep='')]
    df_wide[vn] <- NULL
    df_wide[paste(vn,'_ks', sep='')] <- NULL
  }
}
```

```
#-- we don't split into train/test, as we use LOOCV on full dataset (call it train)

dataset_params <- list(
  feature_pre_filter = FALSE
)

if (ml_model == 'rf') {
  pred_cv = rep(factor(NA, levels = levels(df_wide$subtype_simple)), 0)
}
if (ml_model == 'lgb') {
  pred_cv = rep(NA, 0)
}

tree_imp_cv = list()
cv_boosters_list = list()  # store all nfold boosters as a list

for (fold_index in 1:nrow(df_wide)) {
  # print(fold_index)
  cat(paste(".", fold_index, "."))
  #fold_index = 1  # during debugging

  if (ml_model == 'lgb') {
    # fit model to all observations except fold_index
    # split into training and test set
    train = as.matrix(df_wide[-fold_index, ])
    test = as.matrix(df_wide[fold_index, ])

    # look at distribution of subtype_simple in train and test dataset
    # table(df_wide[-fold_index, ]$subtype_simple)
    # table(df_wide[fold_index, ]$subtype_simple)

    train_x = train[, -which(colnames(train) %in% c('subtype_simple'))]
    train_y = train[, 'subtype_simple']

    test_x = test[, -which(colnames(test) %in% c('subtype_simple')), drop=F]
    test_y = test[, 'subtype_simple', drop=F]

    dtrain = lgb.Dataset(train_x, label = train_y, params = dataset_params)
    dtest = lgb.Dataset.create.valid(dtrain, data = test_x, label = test_y,
                                     params = dataset_params)
  }
  if (ml_model == 'rf') {
    # fit model to all observations except fold_index
    # split into training and test set
    train = df_wide[-fold_index, ]
    test = df_wide[fold_index, ]

    # look at distribution of subtype_simple in train and test dataset
    # table(df_wide[-fold_index, ]$subtype_simple)
    # table(df_wide[fold_index, ]$subtype_simple)

    train_x = train[, -which(colnames(train) %in% c('subtype_simple'))]
    train_y = train[, 'subtype_simple']

    test_x = test[, -which(colnames(test) %in% c('subtype_simple')), drop=F]
    test_y = test[, 'subtype_simple', drop=F]

  }
```

```
  # fit model
  if (ml_model == 'rf') {
    set.seed(71)
    # re OOB confusion matrix and the fact that no cross-validation is required outside
    # rf see Breiman's doc https://www.stat.berkeley.edu/~breiman/RandomForests/cc_home.htm#ooberr
    cv_boosters_list[[fold_index]] <- randomForest(x=train_x,y=train_y,
                                                    importance=TRUE,
                         proximity=TRUE, mtry=rf_nbr_var_sampled_for_each_split, #282,
                         ntree=rf_nbr_trees, # default 500
                         # classwt=c(table(df_wide$subtype_simple)[1] / nrow(df_wide),
                         #           table(df_wide$subtype_simple)[2] / nrow(df_wide)),
                         sampsize=c(rf_sampsize_for_both_classes, rf_sampsize_for_both_classes))

  }
  if (ml_model == 'lgb') {
    # train model
    # re the warning "No further splits with positive gain, best gain: -inf" see
    # https://stackoverflow.com/questions/47770123/lightgbm-how-to-deal-with-no-further-splits-with-positive-gain-best-gain-inf

    cv_boosters_list[[fold_index]] = lgb.train(params = params,
                                               data = dtrain,
                                               nrounds = n_rounds,
    )
  }


  # prediction for fold i
  pred_cv[fold_index] = predict(cv_boosters_list[[fold_index]], test_x, reshape=T)

  if (ml_model == 'rf') {
    tree_imp_cv[[fold_index]] = as.data.frame(round(importance(cv_boosters_list[[fold_index]]), 2))
  }
  if (ml_model == 'lgb') {
    tree_imp_cv[[fold_index]] = lgb.importance(cv_boosters_list[[fold_index]], percentage = T)
  }
}

if (ml_model == 'lgb') {
  prob_threshold <- 0.5
  pred_y_cv = ifelse(pred_cv > prob_threshold, 1,0)
  pred_y_cv = factor(pred_y_cv, levels=min(train_y):max(train_y))
}

if (ml_model == 'rf') {
  pred_y_cv = pred_cv
}

## ---------------------------------------------------------------------------
## Accuracy, visualization of results, feature importance, interactions, etc.
## ---------------------------------------------------------------------------

# accuracy check
if (ml_model == 'rf') {
  confusionMatrix(pred_y_cv, as.factor(df_wide$subtype_simple), positive='one')
}
if (ml_model == 'lgb') {
  confusionMatrix(pred_y_cv, as.factor(df_wide$subtype_simple), positive='1')
```

```
}

if (create_varimportance_plots){
  if (ml_model == 'lgb') {
    for (fold_index in 1:nrow(df_wide)) {
      lgb.plot.importance(tree_imp_cv[[fold_index]], measure = "Gain")
    }

    ## fit again one lgb, using all data now (since before LOOCV, we only have one
    # obs more), i.e. we only have train data
    # explain one single prediction: the last fold_index (35)
    train = as.matrix(df_wide)
    train_x = train[, -which(colnames(train) %in% c('subtype_simple'))]
    train_y = train[, 'subtype_simple']

    dtrain = lgb.Dataset(train_x, label = train_y, params = dataset_params)
    # train model
    # re the warning "No further splits with positive gain, best gain: -inf" see
    # https://stackoverflow.com/questions/47770123/lightgbm-how-to-deal-with-no-further-splits-with-positive-gain-best-gain-inf

    model = lgb.train(params, dtrain, nrounds = n_rounds)

    # feature importance
    lgb.plot.importance(lgb.importance(model, percentage = T), measure = "Gain")

    # shapley values
    shp <- shapviz(model, X_pred = data.matrix(train_x), X = train_x)

    # explaining one single prediction (can be different from the indiv plot above
    # based on cv_boosters_list[[fold_index]])
    sv_waterfall(shp, row_id = 35)
    #sv_force(shp, row_id = 1)

    # explainig the model as a whole
    sv_importance(shp)
    sv_importance(shp, kind = "bar")
    sv_importance(shp, kind = "both", alpha = 0.2, width = 0.2)

  }
  if (ml_model == 'rf') {
    # here we could also just refit one rf, using all data, since varible
    # importance is based on out-of-bag data
    full.rf <- randomForest(subtype_simple ~ ., data=df_wide,
                            importance=TRUE,
                            proximity=TRUE, mtry=rf_nbr_var_sampled_for_each_split,
                            ntree=rf_nbr_trees,
                            sampsize=c(rf_sampsize_for_both_classes,
                                       rf_sampsize_for_both_classes))

    print(full.rf)
    ## Look at variable importance:
    # round(importance(full.rf), 2)
    vimp = as.data.frame(round(importance(full.rf), 2))
    head(vimp[order(vimp['MeanDecreaseAccuracy'], decreasing = T), ])
    head(vimp[order(vimp$MeanDecreaseGini, decreasing = T), ])

    # getting vimp for each fold
    # for (fold_index in 1:nrow(df_wide)) {
```

```
    #   vimp = as.data.frame(round(tree_imp_cv[[fold_index]], 2))
    #   print(head(vimp[order(vimp$MeanDecreaseAccuracy, decreasing = T), ]))
    #   print(head(vimp[order(vimp$MeanDecreaseGini, decreasing = T), ]))
    # }
  }
}
```
