# Supplementary material for: Circulating neutrophils from patients with early breast cancer have distinct subtype-dependent phenotypes
Source: Breast Cancer Res. 2023 Oct 19;25:125. doi: 10.1186/s13058-023-01707-3 (PMC10588170; doi:10.1186/s13058-023-01707-3)
Supplement: Supplementary file 11 — Additional file 11. Figure S6. Kinase activity changes with the stronger weight. [file 13058_2023_1707_MOESM11_ESM.docx]

**Supplementary Figure 6**

**Kinase activity changes with the stronger weight.**

**a.**

**Benign vs Malign**

Variable importance

**one zero MeanDecreaseAccuracy MeanDecreaseGini**

Lck 36.32 37.11 36.75 0.52

PKG1 25.80 27.09 26.79 0.24

PKG2 15.35 15.98 16.14 0.09

p70S6K_br_beta_br_ 9.98 11.76 11.34 0.06

PKC_br_alpha_br_ 5.80 8.10 7.42 0.04

PKG1_ks 6.00 7.41 7.07 0.03

**b.**

**HER2 negative vs HER2 positive**

Variable importance


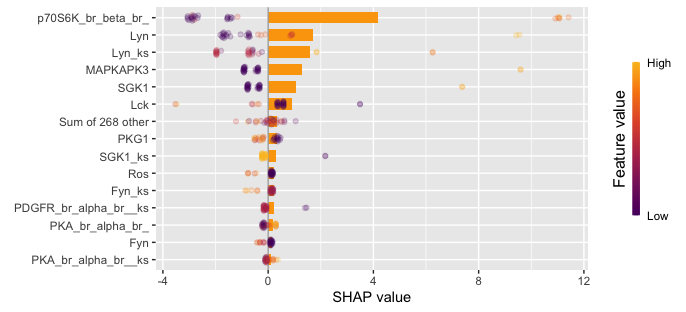


Figure 6a: Differences in kinase activity predicted by model in patients with breast cancer compared with patients with benign breast disease

Figure 6b: Differences in kinase activity in patients with HER2 positive breast cancer compared to patients with HER2 negative breast cancer predicted using the model
