# Supplementary material for: Circulating neutrophils from patients with early breast cancer have distinct subtype-dependent phenotypes
Source: Breast Cancer Res. 2023 Oct 19;25:125. doi: 10.1186/s13058-023-01707-3 (PMC10588170; doi:10.1186/s13058-023-01707-3)
Supplement: Supplementary file 12 — Additional file 12. Figure S7. Upstream Kinase Analysis (UKA) generated list of putative kinases based on peptides targeted for phosphorylation in patients with different breast cancer subtypes. [file 13058_2023_1707_MOESM12_ESM.pdf]

Supplementary Figure 7

Upstream Kinase Analysis (UKA) generated list of putative kinases based on peptides targeted for phosphorylation in patients with different breast cancer subtypes.

Serine Threonine kinase (STK)

HR-pos **HER2 neg** pts Vs HVs

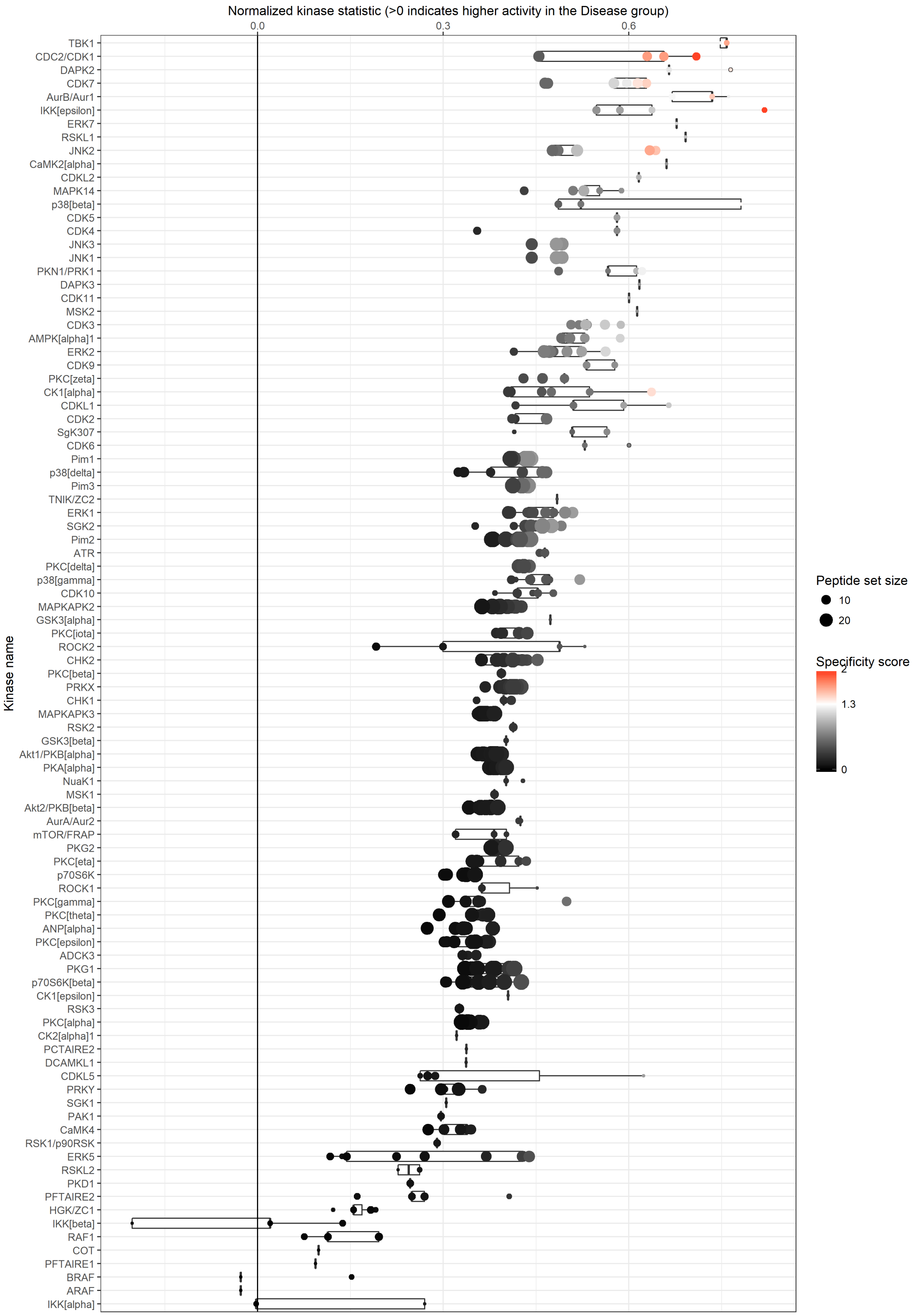

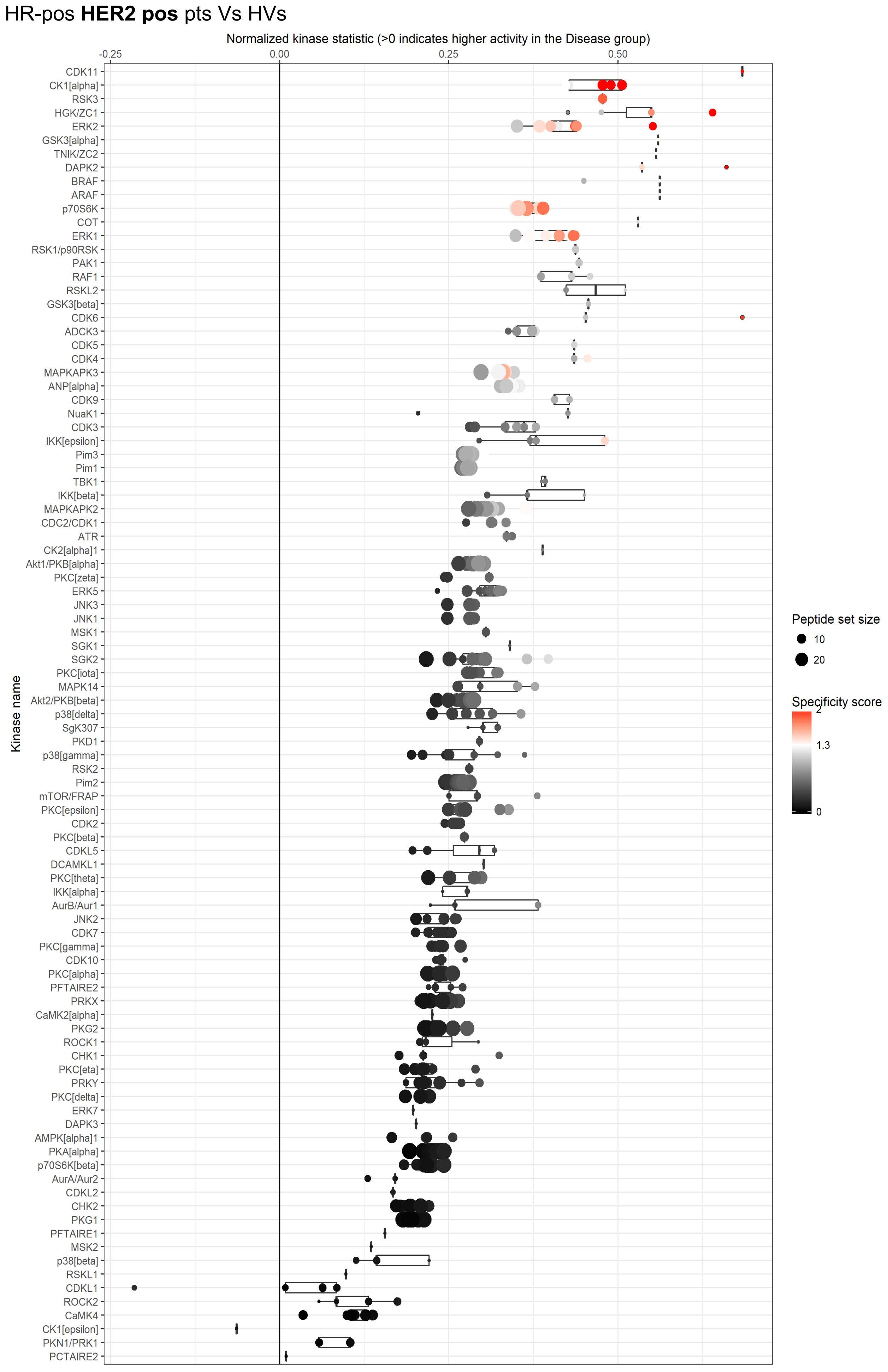

HR-pos **HER2 pos** pts Vs HVs
Normalized kinase statistic (>0 indicates higher activity in the Disease group)
-0.25
0.00
0.25
0.50
Kinase name
CDK11
CK1[alpha]
RSK3
HGK/ZC1
ERK2
GSK3[alpha]
TNIK/ZC2
DAPK2
BRAF
ARAF
p70S6K
COT
ERK1
RSK1/p90RSK
PAK1
RAF1
RSKL2
GSK3[beta]
CDK6
ADCK3
CDK5
CDK4
MAPKAPK3
ANP[alpha]
CDK9
NuaK1
CDK3
IKK[epsilon]
Pim3
Pim1
TBK1
IKK[beta]
MAPKAPK2
CDC2/CDK1
ATR
CK2[alpha]1
Akt1/PKB[alpha]
PKC[zeta]
ERK5
JNK3
JNK1
MSK1
SGK1
SGK2
PKC[iota]
MAPK14
Akt2/PKB[beta]
p38[delta]
SgK307
PKD1
p38[gamma]
RSK2
Pim2
mTOR/FRAP
PKC[epsilon]
CDK2
PKC[beta]
CDKL5
DCAMKL1
PKC[theta]
IKK[alpha]
AurB/Aur1
JNK2
CDK7
PKC[gamma]
CDK10
PKC[alpha]
PFTAIRE2
PRKX
CaMK2[alpha]
PKG2
ROCK1
CHK1
PKC[eta]
PRKY
PKC[delta]
ERK7
DAPK3
AMPK[alpha]1
PKA[alpha]
p70S6K[beta]
AurA/Aur2
CDKL2
CHK2
PKG1
PFTAIRE1
MSK2
p38[beta]
RSKL1
CDKL1
ROCK2
CaMK4
CK1[epsilon]
PKN1/PRK1
PCTAIRE2
Peptide set size
10
20
Specificity score
2
1.3
0

Triple negative pts Vs HVs

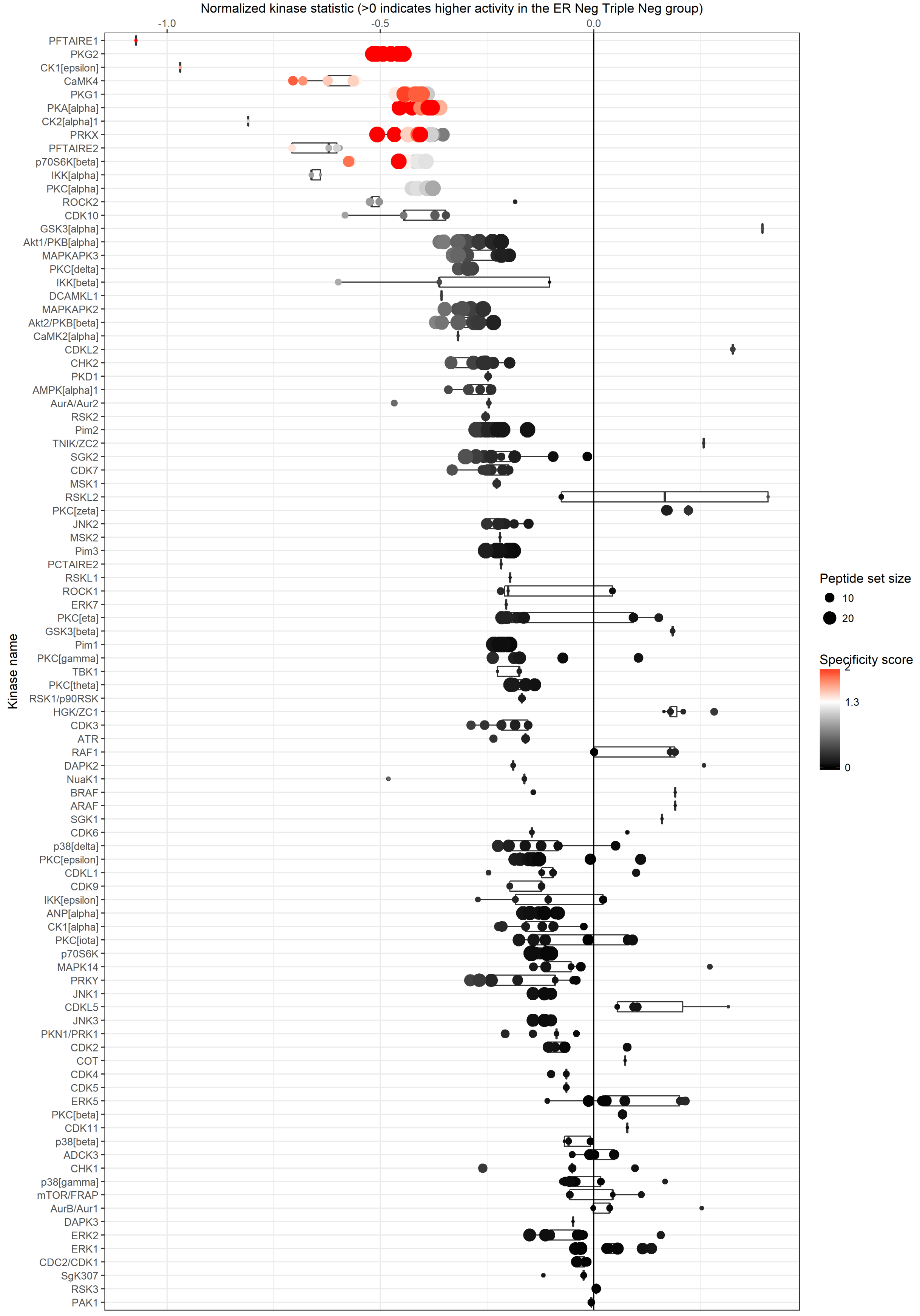

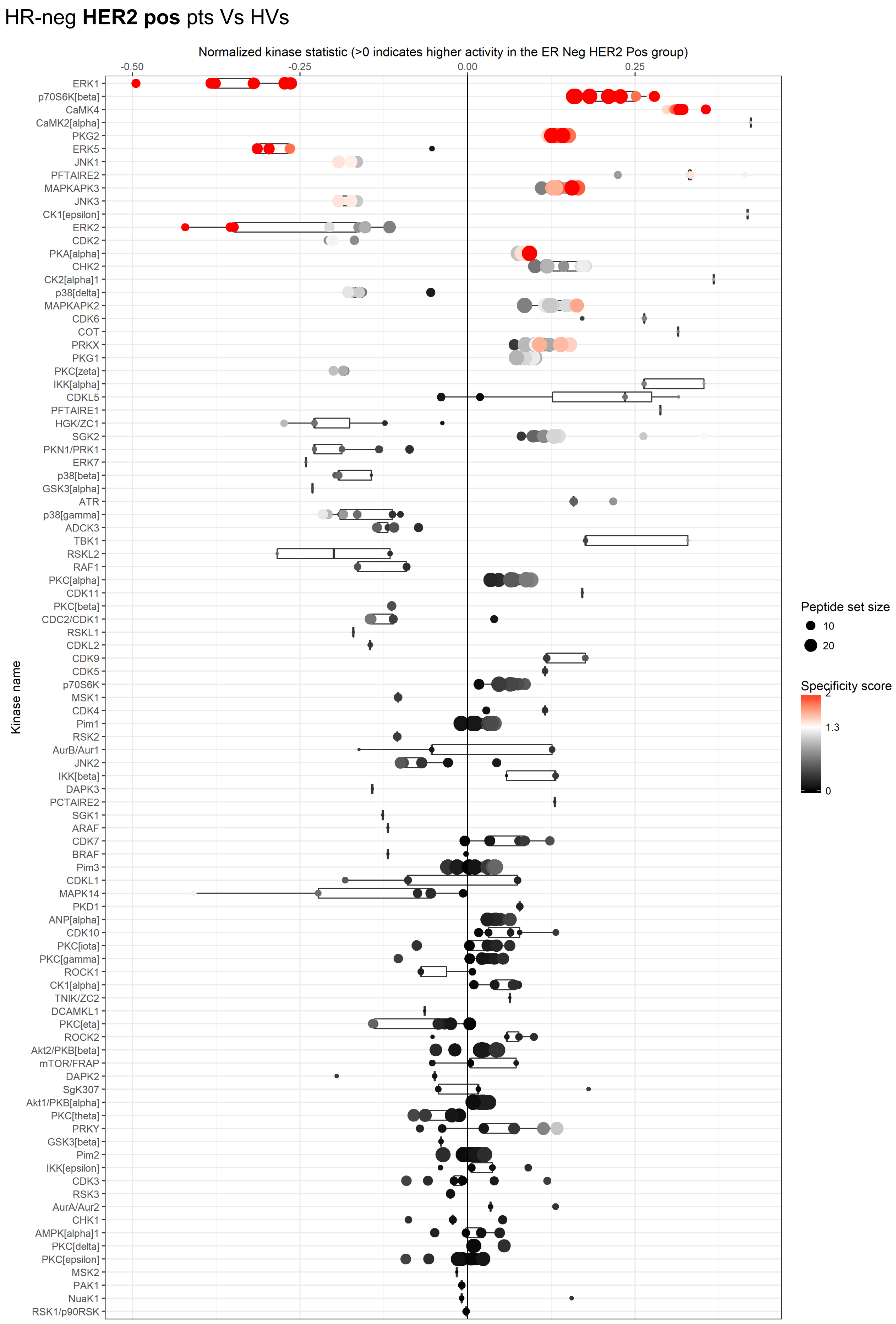

HR-neg **HER2 pos** pts Vs HVs
Normalized kinase statistic (>0 indicates higher activity in the ER Neg HER2 Pos group)
-0.50
-0.25
0.00
0.25
Kinase name
ERK1
p70S6K[beta]
CaMK4
CaMK2[alpha]
PKG2
ERK5
JNK1
PFTAIRE2
MAPKAPK3
JNK3
CK1[epsilon]
ERK2
CDK2
PKA[alpha]
CHK2
CK2[alpha]1
p38[delta]
MAPKAPK2
CDK6
COT
PRKX
PKG1
PKC[zeta]
IKK[alpha]
CDKL5
PFTAIRE1
HGK/ZC1
SGK2
PKN1/PRK1
ERK7
p38[beta]
GSK3[alpha]
ATR
p38[gamma]
ADCK3
TBK1
RSKL2
RAF1
PKC[alpha]
CDK11
PKC[beta]
CDC2/CDK1
RSKL1
CDKL2
CDK9
CDK5
p70S6K
MSK1
CDK4
Pim1
RSK2
AurB/Aur1
JNK2
IKK[beta]
DAPK3
PCTAIRE2
SGK1
ARAF
CDK7
BRAF
Pim3
CDKL1
MAPK14
PKD1
ANP[alpha]
CDK10
PKC[iota]
PKC[gamma]
ROCK1
CK1[alpha]
TNIK/ZC2
DCAMKL1
PKC[eta]
ROCK2
Akt2/PKB[beta]
mTOR/FRAP
DAPK2
SgK307
Akt1/PKB[alpha]
PKC[theta]
PRKY
GSK3[beta]
Pim2
IKK[epsilon]
CDK3
RSK3
AurA/Aur2
CHK1
AMPK[alpha]1
PKC[delta]
PKC[epsilon]
MSK2
PAK1
NuaK1
RSK1/p90RSK
Peptide set size
10
20
Specificity score
2
1.3
0

Phosphotyrosine kinase (PTK)

HR-pos **HER2 neg** pts Vs HVs

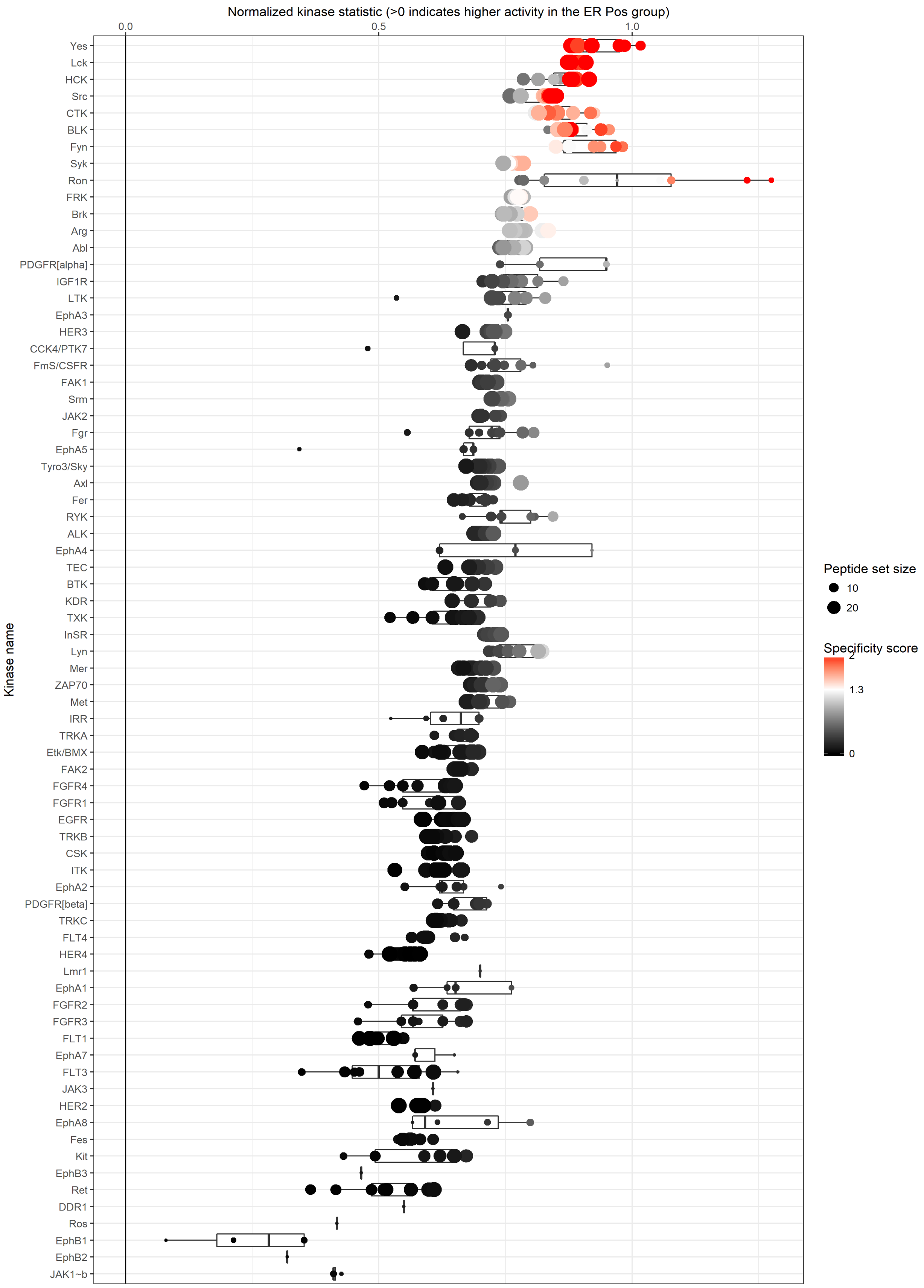

HR-pos **HER2 pos** pts Vs HVs

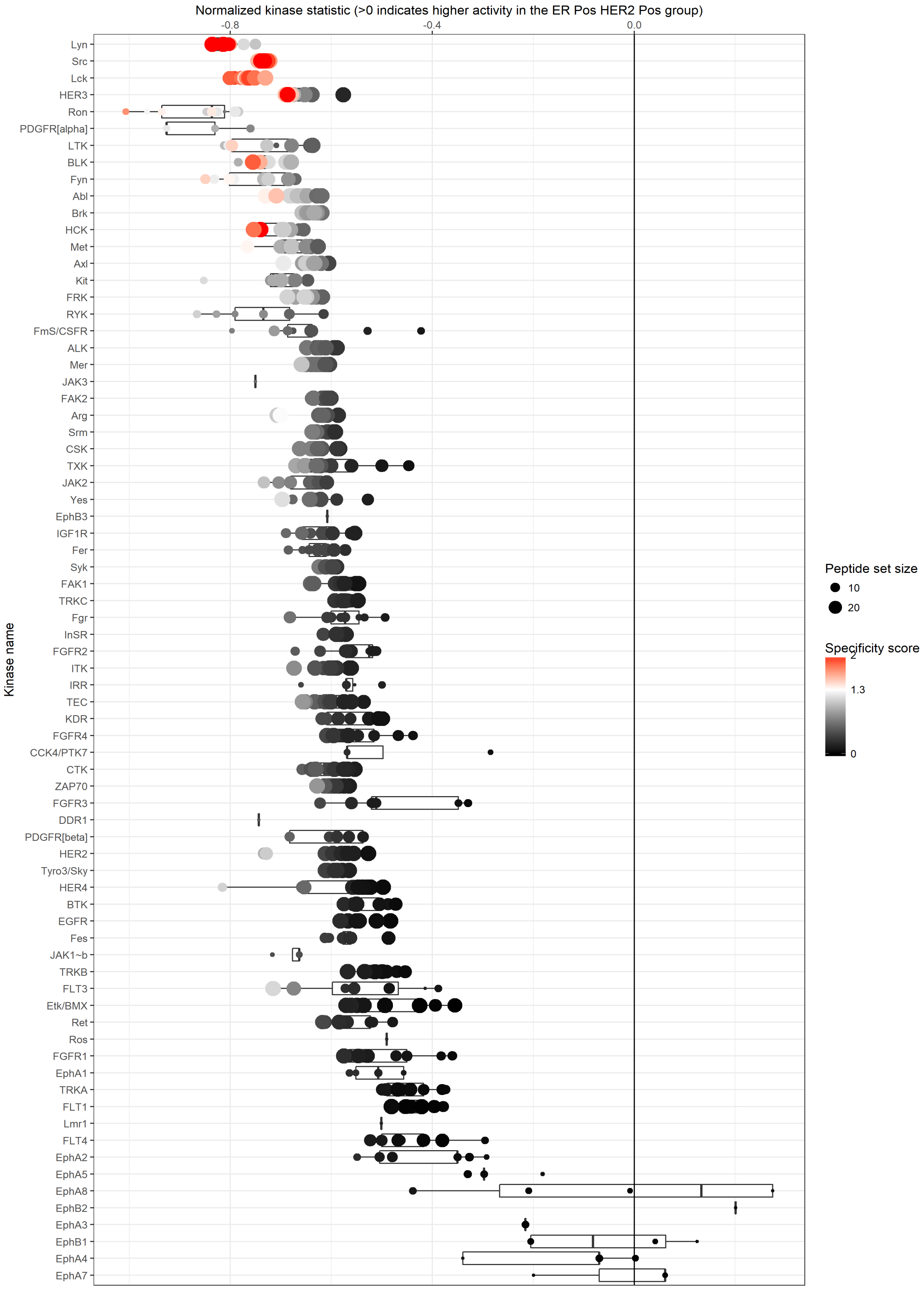

Triple negative pts Vs HVs

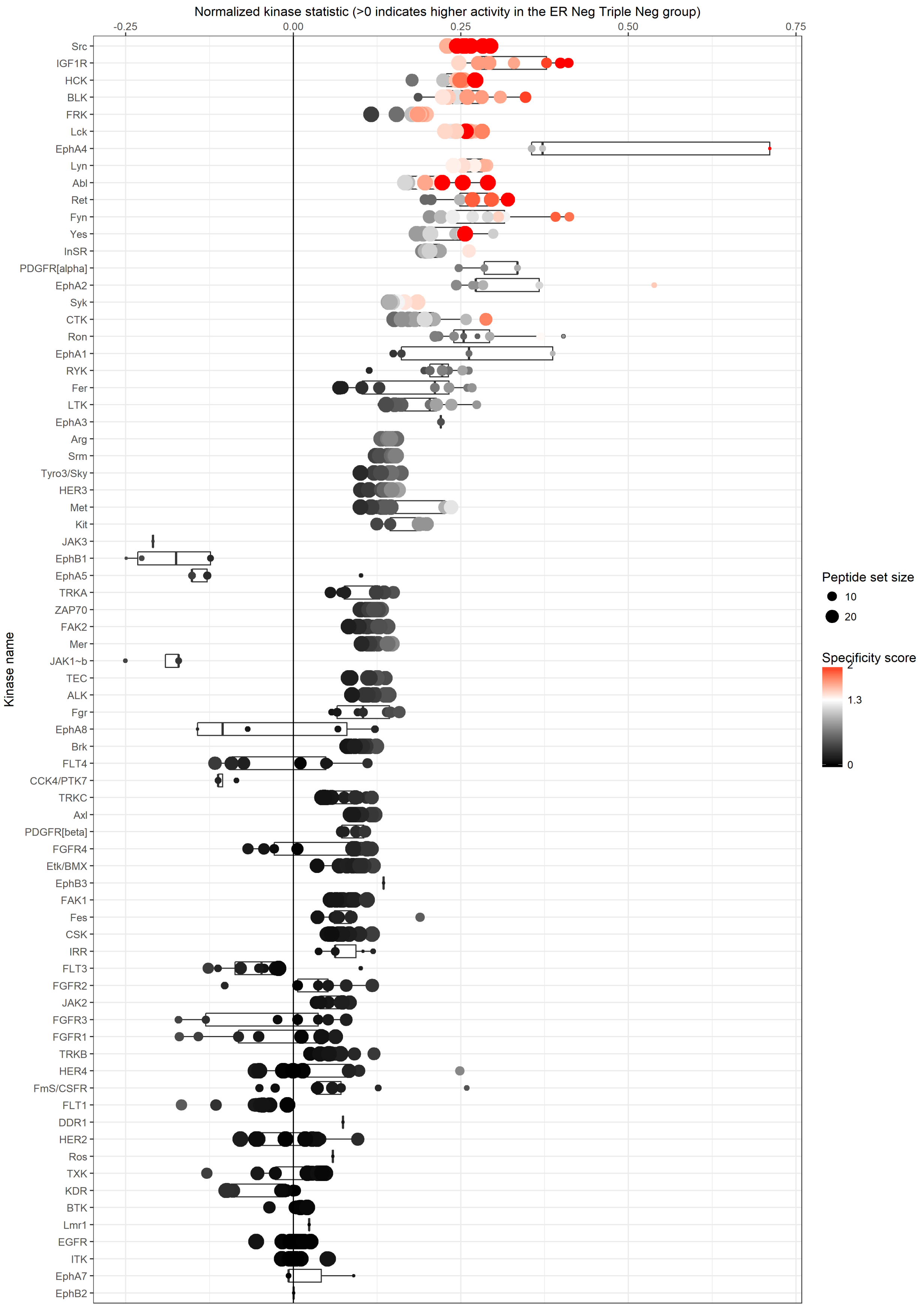

HR-neg **HER2 pos** pts Vs HVs

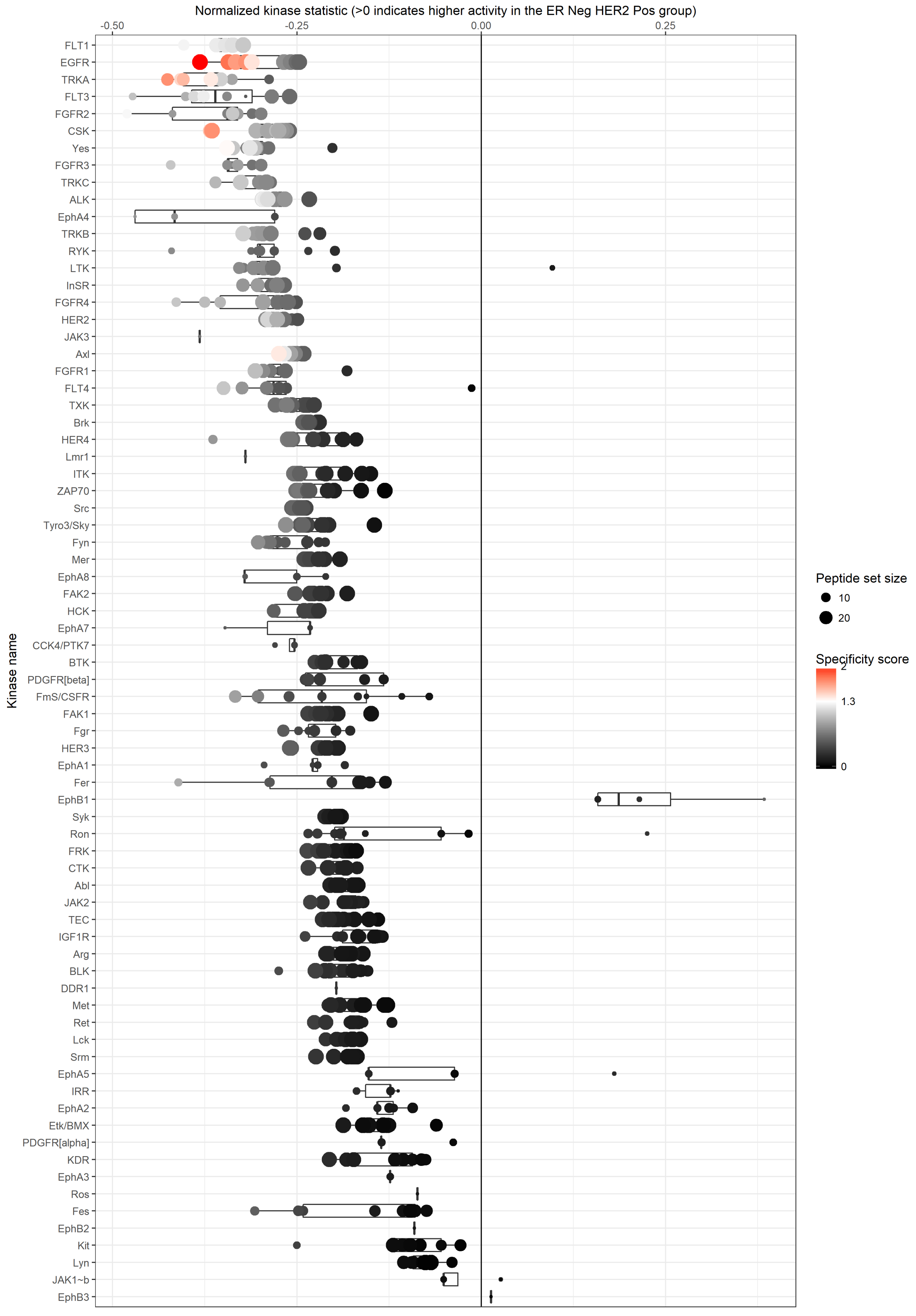

Benign breast disease compared to HVs for **STK family**

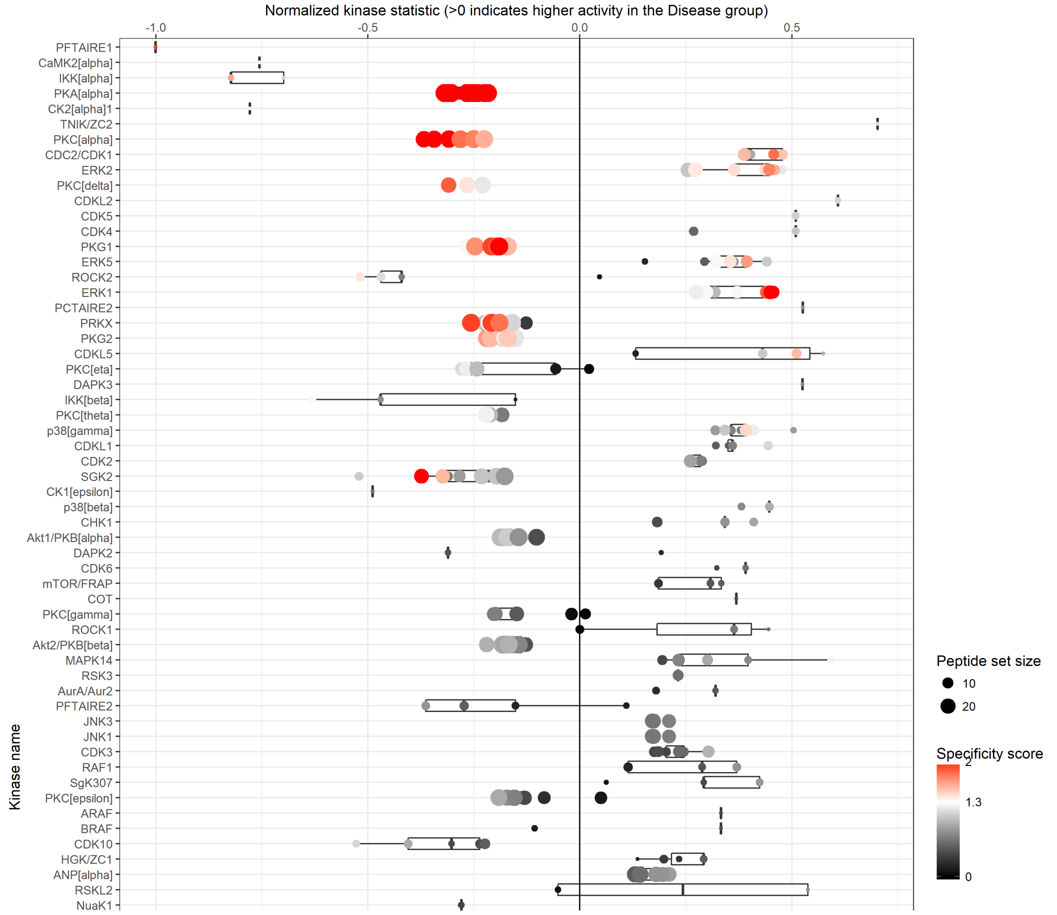

Benign breast disease compared to HVs for **PTK family**

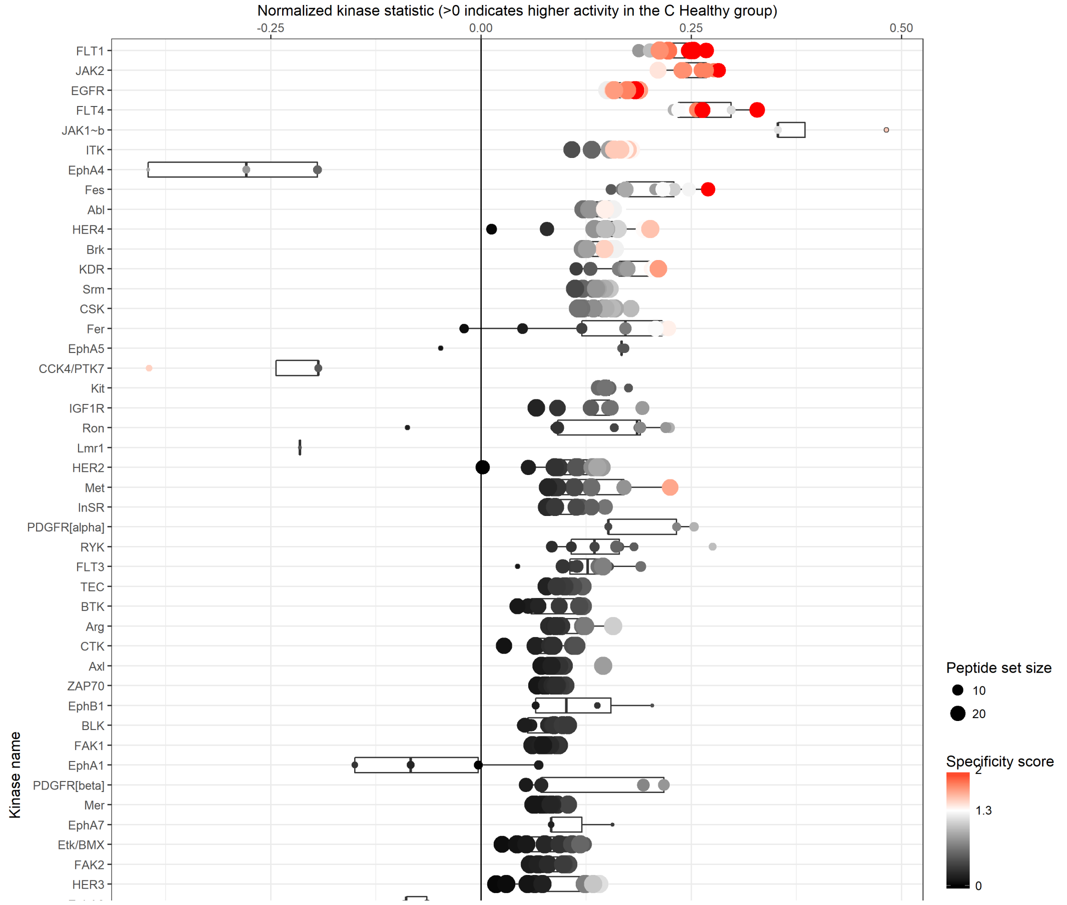

Upstream Kinase Analysis showing a list of putative kinases for either the STK or PTK families based on the peptides phosphorylated differently in patients compared to HVs. The analysis is done for each subtype of breast cancer or patients with benign disease separately as indicated. Size of circle represents peptide data set belonging to each kinase and colour scale represents the specificity of the kinase for that peptide set (red highly specific).
